# Supplementary material for: Biological Function Analysis of MicroRNAs and Proteins in the Cerebrospinal Fluid of Patients with Parkinson’s Disease
Source: Int J Mol Sci. 2024 Dec 10;25(24):13260. doi: 10.3390/ijms252413260 (PMC11678473; doi:10.3390/ijms252413260)
Supplement: Supplementary file 1 [file ijms-25-13260-s001.zip › ijms-3329783-supplementary.pdf]

Supplementary Table S1. List of miRNA symbols identified by Ingenuity Pathway Analysis (IPA) software for 16 miRNAs in miRNAomic network.

No.	IPA symbol	miRBase ID	miRNA symbol	Reference
1	let-7	MI0000063	let-7b	**[87]**
2	miR-1-3p	MIMAT0000462	miR-206	**[52]**
3	mir-126	MI0000471	mir-126	**[86]**
4	miR-126a-5p	MIMAT0000444	miR-126-5p	**[89]**
5	mir-130	MI0000745	mir-301a	**[86]**
6	mir-15	MI0000070	miR-16	**[87]**
7	miR-151-3p	MIMAT0000757	miR-151a-3p	**[52]**
8	miR-151-5p	MIMAT0004697	miR-151a-5p	**[87]**
9	miR-16-5p	MIMAT0000069	miR-16-5p	**[89]**
10	miR-17-5p	MIMAT0000680	miR-106b-5p	**[54]**
11	mir-24	MI0000080	miR-24	**[87]**
12	miR-24-3p	MIMAT0000080	miR-24-3p	**[56]**
13	mir-28	MI0000086	mir-28	**[86]**
14	mir-29	MI0000105	miR-29b-1	**[87]**
15	mir-30	MI0000441	mir-30b	**[86,87]**
16	miR-7a-5p	MIMAT0000252	miR-7-5p	**[54]**
